# Investigation of the multifunctional gene *AOP3* expands the regulatory network fine-tuning glucosinolate production in *Arabidopsis*

**DOI:** 10.3389/fpls.2015.00762

**Published:** 2015-09-23

**Authors:** Lea M. Jensen, Daniel J. Kliebenstein, Meike Burow

**Affiliations:** ^1^DNRF Center DynaMo, Department of Plant and Environmental Sciences, Faculty of Science, University of CopenhagenFrederiksberg, Denmark; ^2^Copenhagen Plant Science Centre, Department of Plant and Environmental Sciences, Faculty of Science, University of CopenhagenFrederiksberg, Denmark; ^3^Department of Plant Sciences, University of California, DavisDavis, CA, USA

**Keywords:** QTL mapping, regulatory RNA, glucosinolates, natural variation, regulatory networks

## Abstract

Quantitative trait loci (QTL) mapping studies enable identification of loci that are part of regulatory networks controlling various phenotypes. Detailed investigations of genes within these loci are required to ultimately understand the function of individual genes and how they interact with other players in the network. In this study, we use transgenic plants in combination with natural variation to investigate the regulatory role of the *AOP3* gene found in *GS-AOP* locus previously suggested to contribute to the regulation of glucosinolate defense compounds. Phenotypic analysis and QTL mapping in F2 populations with different *AOP3* transgenes support that the enzymatic function and the *AOP3* RNA both play a significant role in controlling glucosinolate accumulation. Furthermore, we find different loci interacting with either the enzymatic activity or the RNA of *AOP3* and thereby extend the regulatory network controlling glucosinolate accumulation.

## Introduction

Plants frequently rely upon genetic variation to optimize their fitness across many different environments they cannot escape from. Variation between local environments has resulted in large genomic and phenotypic variation among plants of the same species. Genomic variation can affect any level of regulatory networks and leads to phenotypic variation between accessions for optimization of survival, when present in environments with specific biotic and abiotic challenges (Juenger et al., 2006, 2010; Kliebenstein et al., 2006; Van Leeuwen et al., 2007; Burow et al., 2010; Paul-Victor et al., 2010; Woods et al., 2012).

Genetic variation in regulatory networks greatly complicates our ability to understand how individual genes behave in the context of a species as we often study a single genotype. This means that to understand a gene, even more a pathway, requires studies involving numerous accessions to sample a broad array of the existing network connections. Studying network variation is especially critical considering that variation in these connections may create or change feedback mechanisms and the network's signaling properties. A further complication is the potential for individual genes to have multiple functions that may vary depending upon the specific molecular level. For example, an enzyme-encoding gene could have different functions linked to its enzymatic activity, RNA, substrate or product metabolites (Chooniedass-Kothari et al., 2004; Kloc et al., 2005; Hashimoto et al., 2009; Heo and Sung, 2011). Thus, to understand a pathway and the regulatory network controlling it across accessions requires us to understand the specific molecular basis of variation within individual genes and how their molecular function might change dependent on the genetic state of other polymorphic loci within the species. The function of any gene depends on the accession-specific sequence and expression as well as the polymorphic state of the regulatory network that controls the ultimate phenotype.

Extensive knowledge of natural variants of *Arabidopsis thaliana* allows for studying the link between genetic and phenotypic variation (Borevitz et al., 2007; Atwell et al., 2010; Salomé et al., 2011; Weigel, 2012). One of the more well-studied naturally variable pathways in *Arabidopsis* is the synthesis of the defense compounds glucosinolates (Sønderby et al., 2010; Jensen et al., 2014). Glucosinolates show extensive variation among accessions to provide protection against a large diversity of natural enemies (Kliebenstein et al., 2001b; Burow et al., 2010). In *Arabidopsis*, about 40 glucosinolates are mainly produced from methionine or tryptophan resulting in aliphatic and indolic glucosinolates. Especially the aliphatic glucosinolates derived from methionine display high structural diversity due to variation in chain length and secondary modifications. Nearly all enzymes and several regulators of the pathways have been identified (Sønderby et al., 2010). Thus, the pathway enables detailed investigations of the regulatory function of a gene, which is not a classical transcriptional factor and which might function dependent on the genetic network.

**Figure 1:**
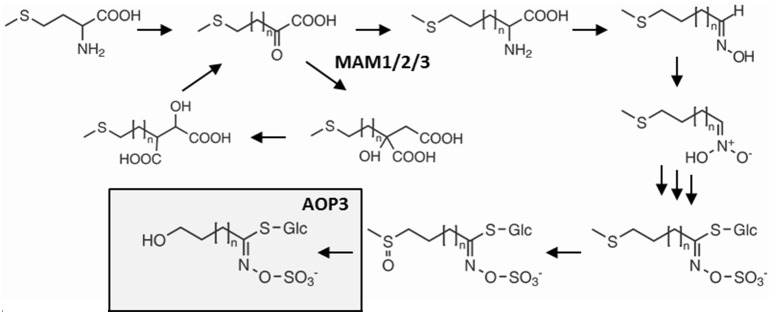
**Overview of the glucosinolate pathway and the enzymatic function of *AOP3***. Selected genes and intermediates in aliphatic glucosinolate biosynthesis from methionine. Dependent on the status of *GS-ELONG* containing the different MAMs, methionine undergoes 1–6 chain elongation cycles before entering the core glucosinolate structure pathway. AOP3 catalyzes production of hydroxyalkyl glucosinolate from C3 side chained methylsulfinylalkyl glucosinolate.

One glucosinolate gene that appears to have extensive variation in its function depending upon the background variation is the glucosinolate biosynthetic gene *AOP3* encoding a 2-oxoglutarate-dependent dioxygenase modifying glucosinolate side chains (Figure 1) (Mithen et al., 1995; Kliebenstein et al., 2001b,c). In addition to its enzymatic function, *AOP3* is associated with an apparent regulatory control of aliphatic glucosinolate accumulation. Introgression lines and natural variation show that *AOP3* increases glucosinolate accumulation compared to the *AOP*^*null*^ allele (Kliebenstein et al., 2001b; Rohr et al., 2009, 2012). A paralogous enzyme, *AOP2*, also has the ability to alter aliphatic glucosinolate levels (Mithen et al., 1995; Kliebenstein et al., 2001b; Wentzell et al., 2007; Burow et al., 2015). Introduction of a functional *AOP2* into the *AOP*^*null*^ *MAM1* background, Col-0, demonstrated the large potential of *AOP2* to increase glucosinolate levels via an unknown mechanism (Wentzell et al., 2007). The specific role of *AOP3* in controlling aliphatic glucosinolate accumulation is less well understood, even though more studies suggest a regulatory role (Kliebenstein et al., 2001a,b,c; Wentzell et al., 2007; Rohr et al., 2012).

The *GS-AOP* locus shows epistasis with *GS-ELONG* for accumulation of glucosinolates (Kliebenstein et al., 2001a). *GS-ELONG* has two major natural allelic variants, *MAM1* and *MAM2*, which are mainly responsible for the production of different chain lengths of the predominant short-chained aliphatic (SC) glucosinolates. AOP3 converts the SC glucosinolates with a C3 side chain, which accumulate in high amounts in *MAM2* plants. Furthermore, the *GS-ELONG* locus encodes MAM3, the only MAM enzyme that catalyzes a step in the production of long-chained aliphatic (LC) glucosinolates with up to 8 carbon atoms (Magrath et al., 1994; Mithen et al., 1995; De Quiros et al., 2000; Kroymann et al., 2001, 2003; Textor et al., 2007). Since the glucosinolate profile might be critical for the regulation of the pathway through flux and feedback, the allelic state of *GS-ELONG* may affect any regulatory function of *AOP3*.

In this study, we investigate the regulatory role of *AOP3* by generating different gene versions and introducing them into different backgrounds to gain insight into the regulatory function of *AOP3* and its interaction with the genetic background. Our study reveals that the regulatory role of *AOP3* is invisible in two chosen accessions, but is unveiled upon mixing the genetic backgrounds, leading to the conclusion that the regulatory function is highly depended on other loci. Quantitative trait loci (QTL) mapping revealed a regulatory function of the *AOP3* RNA and enabled us to identify loci required for the regulatory function of *AOP3*. In summary, we conclude that elucidating the molecular function of a potentially adaptive gene is a complex task as the function might greatly vary dependent on natural variation in the genome and an estimation of one universal *in planta* function might not be possible. However, this provides the plant an elegant ability to fine-tune the phenotypic outcome dependent on expression of different regulatory network components for adaption to different environmental challenges.

## Methods

### Generation of expression constructs

Genomic DNA was extracted from leaf tissue using the CTAB method (Clarke, 2009). *AOP3* was cloned from *Landsberg erecta* gDNA using primers designed based on the reference sequence from TAIR (AT4G03050.2). USER-fusion (Nour-Eldin et al., 2006; Geu-Flores et al., 2007) was used to generate the different versions of *AOP3* and insert them into pCAMBIA330035Su (Nour-Eldin et al., 2006) downstream of the CaMV 35s promoter.

Primer used for *AOP3* FL: 5′-ggcttaauATGGGTTCATGCAGTCCTCA-3′ and 5′-GGTTTAAUTTATTTCCCAGCAGAGACGC-3′.

Primers used for *AOP3* NF fragment 1: 5′-GGCTTAAUATGGGTTCATGCAGTCCTCA-3′ and 5′-AAGGCTTuTAgCAGTAgcACTAGGTAAGCCCAAC-3′, fragment 2: 5′-AAAGCCTuAGTGGAATAATTTATCAGC-3′ and 5′-ACCCTTACuCGGgcATACGG-3′ and fragment 3: 5′-AGTAAGGGuAACAGAGAGAAAGAAGACG-3′ and 5′-GGTTTAAUTTATTTCCCAGCAGAGACGC-3′; small letters indicate mutations that lead to changes in the active site based on sequence similarity to other 2-ODDs (Hogan et al., 2000).

Primers for *AOP3* UT fragment 1: 5′-ggcttaauATGGGTTgATGCAGTCCTCA-3′ and 5′-ACCGACCCCuGAAGCTCCgcTGACACTTG-3′ and fragment 2: 5′- AGTAAGGGuAACAGAGAGAAAGAAGACG-3′ and 5′-GGTTTAAUTTATTTCCCAGCAGAGACGC-3′; small letters indicate mutations introduced to remove start codons and introduce a stop codon. All plasmids were verified by sequencing.

### Generation of transgenic plants

Plasmids were transferred into *Agrobacterium tumefaciens* (strain PGV38 c58). Col-0 and Gie-0 accessions were transformed using the floral-dip method (Clough and Bent, 1998). The T1 seeds obtained were harvested and grown to the 4-leaf stage, before selection was performed by repeatedly spraying with 300 μM Basta. Transgenic plants were furthermore confirmed by PCR with primers specific for the pCAMBIA330035Su:*AOP3* constructs. For each *AOP3* transgene, we obtained multiple independent T1 individuals (except *AOP3* FL in Col-0) to account for any position effect or spontaneous mutations.

### Generation of F2 populations

To create the Col-0 × L*er*-0 F2 population, Col-0 and L*er*-0 were grown until flowering stage and L*er*-0 was used to pollinate Col-0. The F1 plants were allowed to set seeds, and subsequently the F2 population was sown and tested for glucosinolate accumulation.

To create the Col-0 × Gie-0 populations, Gie-0 *AOP3* FL6, FL9, UT2, and UT 10 were grown to flowering state along with Col-0 WT. The four Gie-0 lines were crossed to Col-0 WT using Col-0 as the maternal and Gie-0 lines as paternal. The F1 plants were genotyped by PCR to ensure presence of *AOP3* transgene. Seeds from the F1 populations were collected and F1 plants from identical parental lines were pooled before sowing out 200–300 seeds for each of the four populations. Two hundred and one plants from the FL6 population, 218 from the FL9 population, 151 plants from the UT2 population and 139 plants from the UT10 population were genotyped, phenotyped and subsequently used for QTL mapping. Leaf material for glucosinolate analysis was harvested 29–30 days after sowing.

### Plant growth

For all experiments, seeds were sown in a randomized design and cold stratified at 4°C for at least 2 days before being moved to Percival growth chambers or walk-in chambers. Plants were grown at 80–120 μE/(m^2*^ s), 16 h light, 20°C, 70% relative humidity.

### Analysis of glucosinolate content

Glucosinolates were extracted from a weighed mature fresh leaf or pool of leaves harvested, when leaves were fully expanded. Glucosinolates were extracted with minor modifications from a previously described protocol (Kliebenstein et al., 2001b). Samples were run on an Agilent HP1200 Series HPLC instrument equipped with a C18 column: Supelcosil LC-18-DB, 25 cm × 4.6 mm, 5 μm particle size (Supelco, Bellefonte, PA, USA) or ZORBAX SB-Aq, 25 cm × 4.6 mm, 5 μm particle size (Agilent Technologies) with the following gradient used: 1.5–7% B (5 min), 7–25% (6 min), 25–80% (4 min), 80% B (3 min), 80–35% B (2 min), 35–1.5% B (2 min), and 1.5% B (3 min), flow rate of 1 mL min–1, *A* = H_2_O, *B* = ACN. The eluent was monitored by diode array detection between 200 and 400 nm (2 nm interval). Desulfoglucosinolates were identified based on comparison of retention times and UV absorption spectra with those of known standards (Reichelt et al., 2002). Results are given as nmol/(mg fresh weight) calculated relative to response factors (Fiebig and Arens, 1992; Brown et al., 2003). The individual glucosinolates were grouped as sums based on the biosynthetic pathway.

### Statistics

R version 3.0.1 (2013-05-16) was used for statistical analysis (Team, 2013). To test for significance of glucosinolate locus variation with glucosinolate accumulation in the F2 population we used the lm function for the following linear model GLS = *GS-ELONG* + *AOP3* + *GS-ELONG*:*AOP3* and following the an analysis-of-variance tables was created to find significantly altered mean of a trait using the Anova function from the car package (Fox and Weisberg, 2011). The beanplots were generated using the beanplot package (Kampstra, 2008). For the Col-0 × Gie-0 F2 populations, new models were made based upon the QTL mapping (Table S1) and analyzed with the lm and Anova function as above. For the WT and insertion lines significance was tested using the lm and Anova function for the following linear model GLS = Experiment + Genotype + insertion line nested within Genotype + Experiment:Genotype with specific differences tested *post-hoc* using the pairwise.t.test function with a Holm-adjustment. Summary statistics was found using the SummaryBy from the doBy package (Højsgaard and Halekoh, 2014). For the density plots showing the differences in C3/(C3 + C4) we used the density function from the base package (Team, 2013).

### Genotyping by MassArray

The DNA provided for genotyping was extracted using Qiagen DNeasy 96 Plant Kit according to protocol. Based on the 1001 genome database 100 SNPs polymorphic between Col-0 and Gie-0 was chosen for Sequenom MassARRAY®. The SNPs had been chosen to get full coverage of the genome; however, some SNPs were dropped due to assay problems, thus, 90 SNPs were used for the FL6, 93 SNPs for the FL9 and UT2, and 94 SNPs for the UT10 population. The SNPs were used to generate genetic maps for each mapping population using the Haldane function (Tables S9–S12). The genetic maps were plotted against the physical maps to check for variation in local recombination rates. The plots show some variation in recombination rates with especially lower recombination rates around the centromeres and higher rates in the end of the chromosomes. However, we found the variation within the range of what can be expected (Horton et al., 2012; Salome et al., 2012). Similarly, there was no evidence of segregation distortion except for the noted instance in the FL6 population.

### QTL mapping

Glucosinolate concentrations and ratios for all lines in the four different populations were used for QTL mapping. Windows QTL Cartographer Version 2.5 was used for composite interval mapping determining significant thresholds for each trait by doing 1000 permutations to estimate the 0.05 significance levels (Wang et al., 2012). The main-effect markers were validated in a combined model and tested for Two-Way epistatic interactions using type II ANOVAs in R version 3.0.1 (2013-05-16) using the most significant marker for each QTL.

### RT-PCR

For each genotype four pools of seedlings were grown, three pools from a positive F2 plant and one pool from a F2 plant segregated without the transgene. RNA was extracted from seedlings with Sigma Spectrum Plant Total RNA kit, treated with Sigma DNAse1 and reverse transcribed with iScript (Bio-rad). The *AOP3* transcript was amplified with primer also used for cloning the *AOP3* FL: 5′-ggcttaauATGGGTTCATGCAGTCCTCA-3′ and 5′-ggtttaauTTATTTCCCAGCAGAGACGC-3′. The PCR used 25 cycles with 96°C for 30 s, 56°C for 60 s, and 65°C for 60 s. For the control primers binding actin was used: 5′-ACATTGTGCTCAGTGGTGGA-3′ and 5′-TCATACTCGGCCTTGGAGAT-3′. The PCR used 30 cycles for the same program as above.

## Results

### *AOP3* interacts with *GS-ELONG* to control glucosinolate levels

We started out by crossing Col-0 and L*er*-0 to generate an F2 population to analyze the impact of *GS-ELONG* and *AOP3* on glucosinolate profiles in a mixed genetic background. The epistatic interaction between *GS-ELONG* and *AOP3* was first reported in a Col-0 × L*er*-0 population, however, the allele specific interaction has not been addressed (Kliebenstein et al., 2001a). *GS-ELONG* varies between Col-0 and L*er*-0 resulting in predominant short-chained (SC) aliphatic glucosinolates with either 4-carbon atoms (C4, Col-0) or 3-carbon atoms (C3, L*er*-0). As Col-0 is an *AOP*^*null*^ accession and L*er*-0 an *AOP3* accession, none of the lines express an enzymatically functional *AOP2*, allowing us to focus on the effect of *AOP3*.

Based on their glucosinolate profiles, we divided the plants in the F2 population into four groups. The accumulation of the AOP3 product allowed us to classify plants as expressing AOP3 or not, whereas the ratio of C3/(C3 + C4) indicated the allelic state of the *GS-ELONG* locus. We calculated the ratio in published accessions (Kliebenstein et al., 2001b; Kroymann et al., 2003), which showed that accessions with no functional *MAM1* but a functional *MAM2* at *GS-ELONG* accumulate more than 90% C3, whereas plants expressing a *MAM1* accumulate less than 40%. In agreement with the previously reported higher accumulation of C4 than C3 due to a dominant function of *MAM1* over *MAM2* (Kroymann et al., 2003), our Col-0 × L*er*-0 F2 population did not contain plants accumulating 50–90% C3. Then, we used the 90% threshold to split the populations into C3, corresponding to the absence of a functional *MAM1* allele, and C4, corresponding to the presence of at least one functional *MAM1* allele.

**Figure 2:**
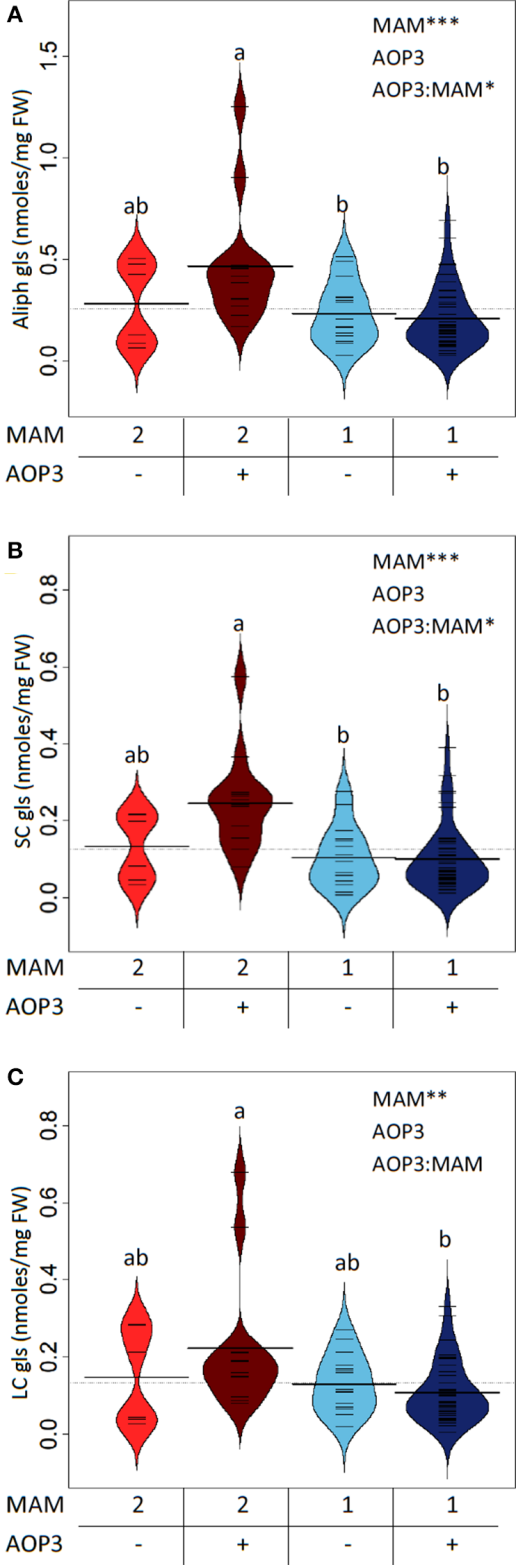
***GS-ELONG* and *AOP3* interaction in a Col-0 × L*er*-0 F2 population for control of leaf glucosinolate levels**. Average glucosinolate levels for the interaction of *MAM1* or *MAM2* with presence and absence of *AOP3* for total aliphatic glucosinolate levels **(A)**, SC glucosinolate levels **(B)**, and LC glucosinolate levels **(C)**. Significance of the main effects and interaction are depicted by *P* < 0.05^*^, *P* < 0.01^**^, and *P* < 0.001^***^ and letters indicate significance of posttest with a levels of *P* < 0.05. Bean plots show strip charts of the individual plants levels in each group (*n* = 6, *n* = 16, *n* = 13, and *n* = 47), the density, and the average.

In the Col-0 × L*er*-0 F2 population, the C3 background has higher levels of total aliphatic glucosinolates than the C4 background i.e., with presence of *MAM1* (Figure 2A). Similarly, plants expressing *AOP3* in the C3 background showed a trend toward higher levels of aliphatic glucosinolates than C3 plants without *AOP3*, whereas no clear difference was seen in the C4 background (Figure 2A, Table S1). This suggests that specific allelic interactions of *GS-ELONG* controlling the relative C3 and C4 accumulation and *AOP3* might play a role in controlling the levels of aliphatic glucosinolates. AOP3 only converts SC glucosinolates, and we therefore considered whether the changes in total aliphatic glucosinolates were purely caused by higher flux for SC. As we expected, the SC levels varied dependent on *AOP3* and *GS-ELONG* as seen for the total aliphatic glucosinolate amounts (Figure 2B), but this was also observed for LC, where the plants with the highest LC levels were seen in the *AOP3 MAM2* background (Figure 2C). These observations suggest that the interaction between *AOP3* and C3 accumulation is important for the fine-tuning of the aliphatic glucosinolate levels, and that *AOP3* is dependent on a homozygous *MAM2* state. Although, the data suggest the importance of the presence of the AOP3 substrate, 3-methylsulfinylpropyl glucosinolate (3msp) (Figure 1), it is not only the flux through the SC pathway that increases the total aliphatic glucosinolate accumulation. Since the increase was also observed for LC glucosinolates that are not substrates for AOP3, the flux needs to be increased from primary to specialized metabolism to cause higher levels of both SC and LC glucosinolates. A potential regulatory role independent of the enzymatic activity of *AOP3* might thus be mediated via signaling by metabolites, protein interactions, or the RNA.

### The regulatory effect of *AOP3* on glucosinolate accumulation requires loci other than *GS-ELONG*

**Figure 3:**
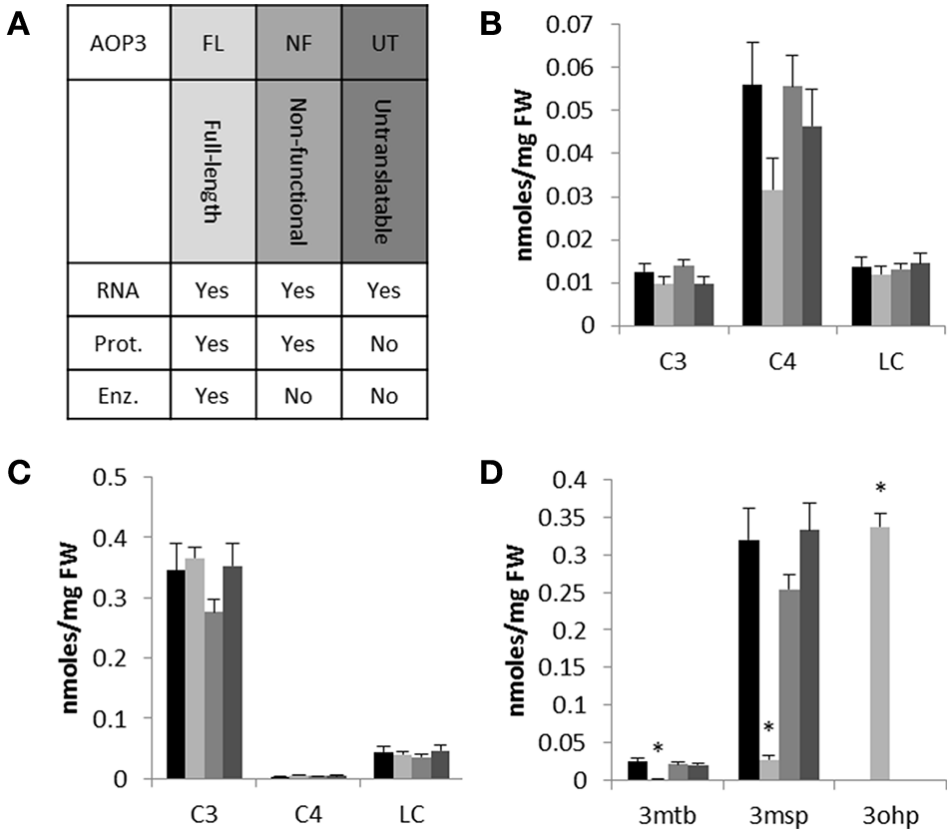
**The effect of introducing different versions of *AOP3* into Col-0 and Gie-0. (A)** Overview of the different *AOP3* constructs for expression *in planta* and their potential to give rise to *AOP3* RNA, protein (Prot.), and enzymatic activity (Enz.). **(B)** Aliphatic glucosinolates with different chain-lengths; C3, C4, and LC. Col-0 WT (black), Col-0 *AOP3* FL (light gray), Col-0 *AOP3* NF (medium gray), or Col-0 *AOP3* UT (dark gray). **(C)** Gie-0 WT (black), Gie-0 *AOP3* FL (light gray), Gie-0 *AOP3* NF (medium gray), or Gie-0 *AOP3* UT (dark gray). **(D)** 3mtp, 3msp, and 3ohp levels in Gie-0 WT (black), Gie-0 *AOP3* FL (light gray), Gie-0 *AOP3* NF (medium gray), or Gie-0 *AOP3* UT (dark gray). Data represent means and standard error for lines carrying the same construct. Differences were tested for significance by ANOVA, ^*^ indicates *P* < 0.05, for additional information see methods and Supplementary Data. FW, fresh weight.

To focus on the effects of *AOP3* and simultaneously test if its regulatory function solely requires the enzymatic activity, we introduced three different versions of the gene into two accessions not expressing a functional *AOP2* or *AOP3* in leaves and varying in the accumulation of C3 and C4 glucosinolates. We chose Col-0 and Gie-0 based on their difference in their major SC glucosinolate, i.e., Col-0 accumulating C4 due to expression of a functional MAM1 and Gie-0 accumulating the AOP3 substrate, 3msp, due to MAM2 expression. We introduced different versions of *AOP3* driven by a 35S promoter (Figure 3A). Accessions that express *AOP3* in leaves use the *AOP2* promoter that has previously been shown to be at least as strong as the 35S promoter (Wentzell et al., 2007; Chan et al., 2010; Kerwin et al., 2011). In addition to an enzymatically functional full-length genomic version of *AOP3*, we constructed a version with a mutation abolishing the active site of AOP3 (Hogan et al., 2000), which generates a non-active enzyme but still expresses a protein allowing us to test for the importance of the enzymatic activity. Generation of the third version included introduction of a stop codon as the third codon of the transcript and chancing the subsequent two potential start codons in frame, i.e., a construct that is unable to generate any AOP3 protein but only the transcript enabling us to test the function of the *AOP3* RNA. Together, these three different versions of *AOP3* allowed us to systematically test whether its regulatory capacity in any of the two accessions varying in *GS-ELONG* relies on its enzymatic activity, the protein, or the RNA (Figure 3A).

We genotyped plants for the transgene and measured glucosinolate accumulation in independent insertion lines. In the Col-0 background, we were only able to include one line expressing the enzymatically active AOP3 (FL); neither this line not the three lines expressing the non-functional (NF) or the three lines expressing the untranslatable *AOP3* (UT) accumulated levels of C3, C4, or LC glucosinolates significantly different from Col-0 WT (Figure 3B, Table S2). Similarly, for three independent insertion lines of each construct in Gie-0, we did not observe any changes in C3, C4, or LC levels compared to Gie-0 WT (Figure 3C, Table S3), although the plants expressing the active AOP3 accumulated high levels of the AOP3 product 3-hydroxypropyl glucosinolate, (3ohp) (Figures 1, 3D). This suggests that in contrast to the Col-0 × L*er*-0 F2 population, *AOP3* does not have an ability to change total levels of C3, C4, or LC glucosinolate levels in the Col-0 or the Gie-0 background. Thus, the previously suggested regulatory role of *AOP3* is not solely dependent on the allelic status at *GS-ELONG*, but instead, there are additional loci being contributed from other accessions that control this effect. Consequently, we cannot conclude on the regulatory entity of *AOP3* based on the stable transgenic lines likely as a consequence of the absence of the required background network polymorphisms.

### The *AOP3* enzyme regulates SC glucosinolates in the mixed genetic background in the F2 populations of Col-0 and Gie-0

Our results in combination with previous studies indicated that *AOP3's* ability to control glucosinolate accumulation is highly dependent upon known and unknown background loci that vary across natural accessions (Kliebenstein et al., 2001a,b; Rohr et al., 2012). Thus, to test the regulatory effect of *AOP3* as an enzyme and as an RNA in a mixed genetic background of Col-0 and Gie-0, several crosses were generated. We crossed the Col-0 WT with two independent Gie-0 lines expressing the functional AOP3 (FL6 and FL9) and two independent Gie-0 lines containing the untranslatable *AOP3* (UT2 and UT10). Genotyping of the F1 progeny from these crosses identified plants positive for the insertion, which were then allowed to self. The subsequent F2 populations will have randomized background loci of the two parents including presence and absence of transgene. This allows us to test the effect of the different *AOP3* constructs in the different segregating backgrounds.

**Figure 4:**
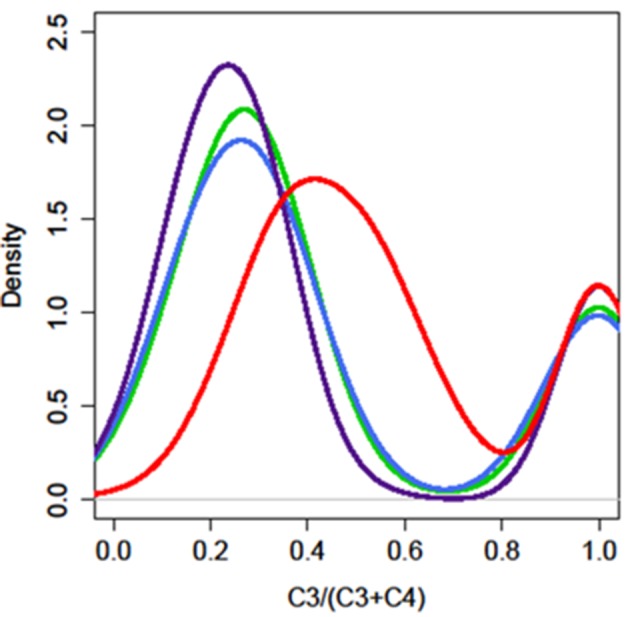
**The enzymatic activity of AOP3 causes a shift in the production of C3–C4 glucosinolates**. The distribution of plants for different C3/(C3 + C4) ratios in the four different *AOP3* populations. Red = *AOP3* FL6 population, green = *AOP3* FL9 population, purple = *AOP3* UT2 population, and blue = *AOP3* UT10 population.

We analyzed the glucosinolate content of 200–300 plants from each F2 population segregating from the four different parents and the expression of *AOP3* in the offspring F3 population (Figure S1, Tables S5–S8). Glucosinolate analysis revealed that in contrast to the FL6 population, none of the plants in the FL9 population contained the product of the AOP3 enzyme, 3ohp, although both the FL6 and FL9 population segregated with the construct encoding the active AOP3 enzyme and expression of the transcript (Figure S1). The absence of 3ohp product shows that the FL9 construct has been functionally silenced by an unknown mechanism. Thus, the FL6 and FL9 population contain the same construct, however, the functional silencing lead to different phenotypic consequences. A survey of the ratio of C3/(C3 + C4) showed that the FL6 population had a shifted ratio in comparison to the FL9 population. Plants with ratios between 0.5 and 0.9 [50–90% C3/(C3 + C4)] were seen to a large extent in the FL6 population containing the enzymatically active AOP3. In contrast, no plants with these ratios were found in the FL9, UT2, and UT10 populations (Figure 4). The plants with high C3/(C3 + C4) ratios from the FL6 population had on average higher levels of 3ohp than the plants with lower relative amounts of C3. Thus, a high level of conversion of 3msp to 3ohp by the active AOP3 mediates a shift from C4 to C3 glucosinolate production showing that the enzymatic activity contributes to regulation of glucosinolate profiles. However, this capacity may depend on other genetic loci specific to Gie-0, as no plants in the Col-0 × L*er*-0 population displayed this unusual C3/(C3 + C4) ratio.

### The RNA encoded by *AOP3* has a regulatory role different from the enzymatic activity

**Figure 5:**
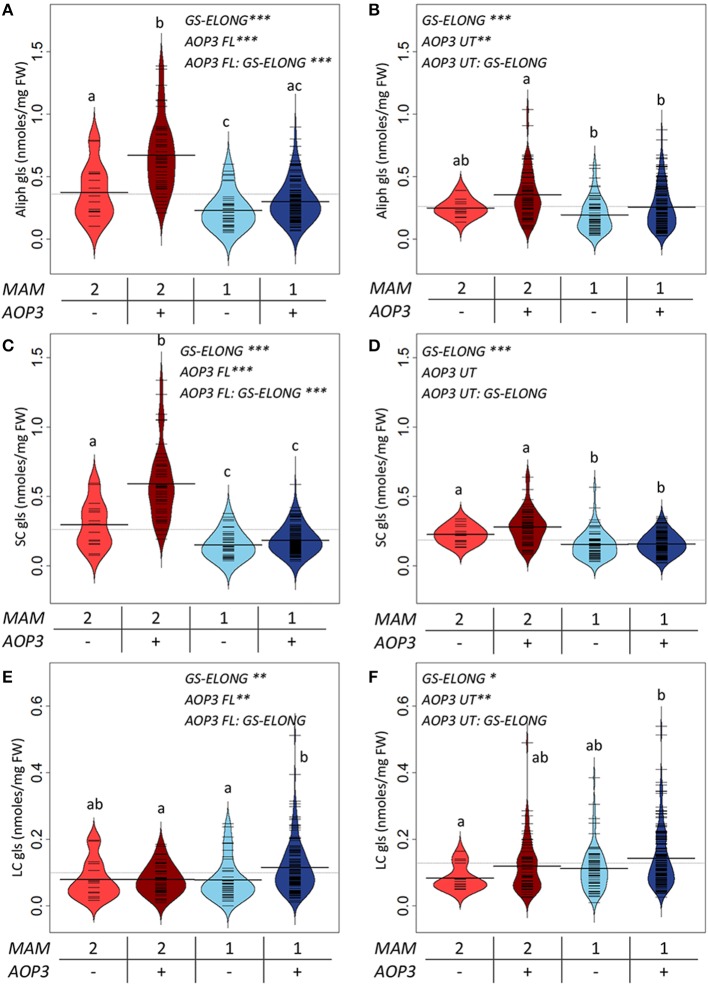
***GS-ELONG* and *AOP3* interaction in Col-0 × Gie-0 F2 populations for control of leaf glucosinolate levels**. Average glucosinolate levels for the interaction of *MAM1* or ***MAM2*** with presence and absence of *AOP3.* For total aliphatic glucosinolate levels with the active AOP3 **(A)** and the *AOP3* RNA **(B)**, for SC glucosinolate levels with the active AOP3 **(C)** and the RNA **(D)**, and for LC glucosinolate levels for the active AOP3 **(E)** and the untranslatable *AOP3* **(F)**. Significance of the main effects and interaction are depicted by *P* < 0.05^*^, *P* < 0.01^**^, and *P* < 0.001^***^ and letters indicate significance of posttest with a levels of *P* < 0.05. Bean plots show strip charts of the individual plants levels, the density, and the average. For the population segregating with the active AOP3 *n* = 16, *n* = 44, *n* = 45, and *n* = 134 for the four groups. For the population segregating with the untranslatable RNA *n* = 14, *n* = 63, *n* = 52, and *n* = 152. FW, fresh weight.

For a more explicit test of the functions of the different *AOP3* constructs and how the gene may be affected by the segregating background, we split the populations based on the lines' *AOP3* transgene status and the accumulation of C3/(C3 + C4) as previously. We then tested if the genotypes at *GS*-*ELONG* (*MAM1* or *MAM2* inferred by the C3/(C3 + C4) threshold) and at *AOP3* are linked with altered glucosinolate levels using the genotypes as factors in a linear model (Figure 5, Table S4). In agreement with previous observations, there was a significant interaction between the presence of the *AOP3* FL construct and the *GS-ELONG* status for total aliphatic glucosinolate accumulation (Figure 5). Interestingly, the untranslatable *AOP3* and the enzymatically active AOP3 had different effects on different glucosinolates suggesting that they influence different parts of the pathway. The functional AOP3 enzyme led to higher SC glucosinolate levels (Figure 5A), but this effect was not significant for the untranslatable *AOP3* (Figure 5B). The higher accumulation of SC glucosinolates was found in plants with the interaction of the active AOP3 and *MAM2* i.e., C3 (Figure 5C). Thus, the enzymatically active AOP3 can regulate SC accumulation, when present in a network containing *MAM2* and other yet unknown components. A similar effect on SC levels was not observed of the *AOP3* RNA (Figure 5D). In contrast, both the active AOP3 and the untranslatable *AOP3* show significant effect on LC glucosinolate accumulation. No significant interaction between AOP3 and *GS-ELONG* was observed for this effect, thus, the effect does not dependent on whether plants produce predominantly C3 or C4 glucosinolates (Figures 5E,F). Together, this suggests that the effect of *AOP3* on LC glucosinolates is not caused by the enzymatic activity and the associated flux, but instead by the *AOP3* RNA in specific genetic backgrounds.

### QTL mapping supports independent regulatory roles for the *AOP3* enzyme and RNA

**Figure 6:**
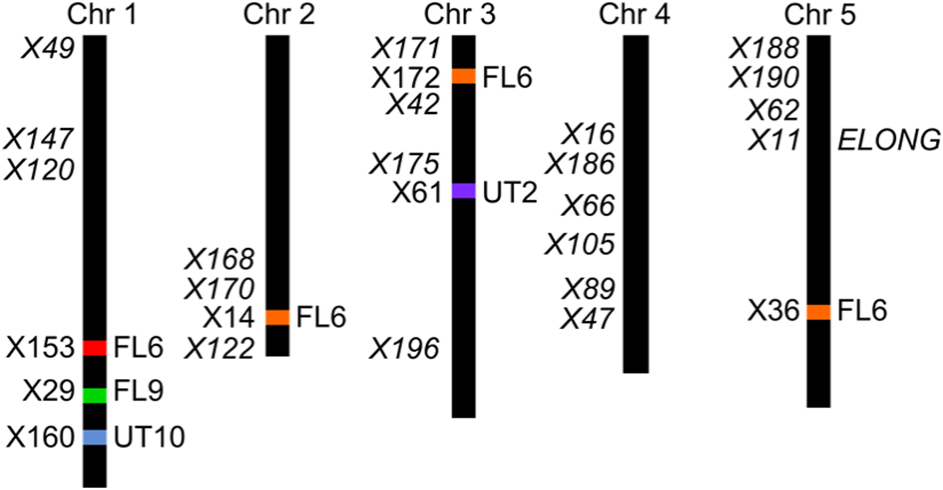
**Position of the transgene(s) in the four populations**. Four different positions were found for the active *AOP3* FL6 (red, orange) by QTL mapping, however, co-segregation of the surrounding regions for the insert on chromosome 2, 3, and 5 (orange), suggests chromosomal rearrangements causing that what looks like three inserts are one found on any of three chromosomes. The *AOP3* FL9 (green) and UT10 (blue) were found at chromosome 1, whereas the *AOP3* UT2 (purple) is positioned on chromosome 3. The closest marker for each insertion site is indicated as well as all significant markers for a phenotype is depicted in italics.

The four F2 populations have randomly shuffled background loci as a result of recombination in the F1 generation. This allows us to use QTL mapping to investigate the effect of the AOP3 enzyme and *AOP3* RNA on glucosinolate accumulation as well as to identify other unidentified loci that vary between the parents and link to glucosinolates. To provide genotype information for QTL mapping, the four populations were genotyped for 100 SNPs using Sequenom MassARRAY® (Tables S9–S12). The physical position of the *AOP3* transgene in each population was found by genotyping the plants for the transgene or based on accumulation of the AOP3 product, 3ohp. Mapping of the *AOP3* transgene in three of the populations (FL9, UT2, and UT10) were in agreement with the presence of a single transgene. In the Col-0 × Gie-0 FL9 F2 population the insertion mapped to chromosome 1, in the UT2 to a position on chromosome 3 and in the UT10 at chromosome 1 (Figure 6). Genotyping of the Col-0 × Gie-0 FL6 population showed the same pattern of SNP markers on parts of chromosome 2, 3, and 5 across the population, which suggests that these chromosome parts co-segregated. This observation can be explained by a chromosomal rearrangement causing the three chromosome parts to be located at one chromosome. We identified these three regions by QTL mapping for the ratio of AOP3 product to C3 glucosinolate, which correlates with the presence of the *AOP3* transgene on all three chromosomes, thus, suggesting chromosomal rearrangement associated with the *AOP3* insertion. We also identified a position on chromosome 1 that contained a second copy of the transgene. For further analysis, we therefore included *AOP3* as a marker for presence, when either position was positive for the insertion.

After identification of the position of the different *AOP3* insertions within each population, we performed QTL mapping and used this information to develop multifactorial linear models for SC, LC, or indole glucosinolates to test the effect of the *AOP3* insertions on all phenotypes in the context of the other genetic loci (Table S1). In the Col-0 × Gie-0 FL6 population, the *AOP3* transgene was significantly linked to alterations in the levels of several individual SC glucosinolates (Table 1). Thus, the active AOP3 is indeed a significant regulator of glucosinolate accumulation. In contrast, it was not possible to identify any significant link between the *AOP3* insertion in the Col-0 × Gie-0 FL9 population and altered glucosinolate accumulation, which may be explained by the functional silencing of the transgene in this population.

**Table 1:** **Significance of AOP3 as a main effect for glucosinolate levels**.

	**AOP3 FL6**	**AOP3 FL9**	**AOP3 UT2**	**AOP3 UT10**
3ohp	**0.025**	–	–	–
3msp	**< 0.001**	0.106	**<0.001**	0.927
3mtp	**< 0.001**	0.600	0.167	0.708
4msb	**< 0.001**	0.426	0.574	0.772
4mtb	0.297	0.442	0.752	**0.042**
7msh	0.417	0.990	0.416	**0.004**
8mso	0.399	0.689	0.137	0.085
I3M	0.358	0.532	0.304	**0.026**
4M−I3M	**0.010**	0.142	0.254	**0.026**
C3	0.096	0.123	**<0.001**	0.936
C4	**<0.001**	0.467	0.550	0.370
SC	0.377	0.564	**0.046**	0.914
LC	0.465	0.768	0.149	**0.042**
Indole	0.297	0.484	0.361	**0.021**

Main effect significance values from models in individual populations. Significance values < 0.05 are depicted in bold.

Mapping for QTLs controlling glucosinolate levels identified loci other than *AOP3* in the Col-0 × Gie-0 FL6 and FL9 populations (Table 2, Figure 6). In both populations a position on chromosome 5 showed up, which corresponds to the *GS-ELONG* locus illustrating its significance. Further, in the population FL6 with the active AOP3, we observed a significant interaction between *AOP3* and *GS-ELONG* for SC glucosinolates (Table 3). This is exclusively observed with the active AOP3 in the FL6 population indicating that this genetic interaction is dependent on the enzymatic activity of *AOP3*. Additionally, we observed significant interactions between the active AOP3 and loci at chromosomes 1 and 3. These may be important for the shifted C3/(C3 + C4) ratio observed in the population.

**Table 2:** **Significance of main effects additional to AOP3 for glucosinolate levels**.

**AOP3 FL6**					**AOP3 FL9**				**AOP3 UT2**				**AOP3 UT10**			
	**Chr**	**Position**	**Marker**	***P*-value**	**Chr**	**Position**	**Marker**	***P*-value**	**Chr**	**Position**	**Marker**	***P*-value**	**Chr**	**Position**	**Marker**	***P*-value**
3ohp	5	4080629	X190	0.023	–	–	–	–	–	–	–	–	–	–	–	–
		7881427	X11 (ELONG)	< 0.001												
3msp	1	28762451	X160	0.021	2	15723768	X170	< 0.001	2	18745766	X122	0.047	5	7881427	X11 (ELONG)	< 0.001
	5	7881427	X11 (ELONG)	< 0.001	5	7881427	X11 (ELONG)	< 0.001	3	1147747	X171	< 0.001				
									4	11326180	X89	0.025				
									5	4080629	X190	< 0.001				
									5	7881427	X11 (ELONG)	0.004				
3mtp	5	7881427	X11 (ELONG)	0.004	5	7881427	X11 (ELONG)	< 0.001	2	18745766	X122	0.027	5	7881427	X11 (ELONG)	< 0.001
									5	4080629	X190	0.024				
4msb	5	7881427	X11 (ELONG)	< 0.001	5	7881427	X11 (ELONG)	< 0.001	4	11326180	X89	0.008	5	7881427	X11 (ELONG)	< 0.001
4mtb	5	7881427	X11 (ELONG)	< 0.001	5	7881427	X11 (ELONG)	0.017	4	11326180	X89	0.001	1	8015459	X120	< 0.001
													3	7313855	X175	0.004
													4	2441219	X16	0.019
													5	7881427	X11(ELONG)	0.010
7msh	4	9213296	X66	< 0.001	3	7313855	X175	0.022								
	5	7881427	X11 (ELONG)	< 0.001	4	13576582	X47	0.032	4	2441219	X16	0.012	5	6708012	X62	< 0.001
					5	7881427	X11 (ELONG)	0.002								
8mso									2	13131014	X168	0.032	3	3679535	X42	0.009
	NS	NS	NS	NS	NS	NS	NS	NS	3	18807903	X196	0.027	5	6708012	X62	< 0.001
									4	2441219	X16	0.028				
I3M					1	1602137	X49	0.003	4	5376168	X186	0.002	4	9580030	X105	0.008
	5	7881427	X11 (ELONG)	< 0.001	1	6375610	X147	0.031	5	1881725	X188	0.014	5	7881427	X11(ELONG)	0.044
					4	11326180	X89	< 0.001	5	7881427	X11(ELONG)	< 0.001				
					5	7881427	X11 (ELONG)	0.002								
4M-I3M	NS	NS	NS	NS	3	3679535	X42	0.023	1	8015459	X120	< 0.001	4	9580030	X105	0.022
					4	11326180	X89	0.005					5	1881725	X188	0.012
C3	5	4080629	X190	0.012	2	15723768	X170	< 0.001	2	18745766	X122	0.025	5	7881427	X11 (ELONG)	< 0.001
	5	7881427	X11 (ELONG)	< 0.001	5	7881427	X11 (ELONG)	< 0.001	3	1147747	X171	< 0.001				
									4	11326180	X89	0.028				
									5	4080629	X190	< 0.001				
									5	7881427	X11 (ELONG)	0.005				
C4	5	7881427	X11 (ELONG)	< 0.001	5	7881427	X11 (ELONG)	0.017	4	11326180	X89	0.001	3	7313855	X175	0.033
													4	2441219	X16	0.049
													5	7881427	X11 (ELONG)	< 0.001
SC	5	4080629	X190	0.030	2	15723768	X170	< 0.001	4	11326180	X89	< 0.001	3	7313855	X175	0.017
	5	7881427	X11 (ELONG)	< 0.001	5	7881427	X11 (ELONG)	< 0.001	5	4080629	X190	0.039	5	7881427	X11 (ELONG)	< 0.001
LC	4	9213296	X66	0.031	NS	NS	NS	NS	3	18807903	X196	0.036	3	3679535	X42	0.016
									4	2441219	X16	0.017	5	6708012	X62	< 0.001
Indole	5	7881427	X11(ELONG)	< 0.001	1	1602137	X49	0.003	4	5376168	X186	0.003	4	9580030	X105	0.006
					1	6375610	X147	0.022	5	1881725	X188	0.019	4	7881427	X11(ELONG)	0.046
					4	11326180	X89	< 0.001	4	7881427	X11(ELONG)	0.001				
					5	7881427	X11(ELONG)	0.001								

**Table 3:** **Estimates of significance of interactions in the different AOP3 population for glucosinolate levels**.

	**AOP3 FL6**		**AOP3 FL9**		**AOP3 UT2**		**AOP3 UT10**	
	**Interactions**	***P*-value**	**Interactions**	***P*-value**	**Interactions**	***P*-value**	**Interactions**	***P*-value**
3ohp	X160:X11(ELONG)	0.010	–	–	–	–	–	–
	X179:X11(ELONG)	0.006						
3msp	**AOP3:X190**	0.002	X120:X11(ELONG)	0.009	**AOP3:X122**	0.004	NS	NS
	**AOP3:X11(ELONG)**	< 0.001	X159:X89	0.016	X122:X89	0.025		
			X159:X11(ELONG)	< 0.001	X122:X112	0.007		
					X89:X11(ELONG)	0.045		
					X89:X171	0.009		
					X89:X112	0.013		
					X190:X11(ELONG)	0.005		
					X171:X11(ELONG)	0.015		
					X190:X112	0.004		
					X171:X112	0.008		
3mtp	NS	NS	NS	NS	**AOP3:X122**	< 0.001	**AOP3:X175**	0.005
					X122:X112	< 0.001		
4msb	**AOP3:X160**	0.004	NS	NS	NS	NS	**AOP3:X53**	0.046
4mtb	X160:X190	0.005	NS	NS	X89:X11(ELONG)	0.028	X120:X53	0.046
	X160:X11(ELONG)	0.033					X120:X11(ELONG)	0.044
							X175:X53	0.018
7msh	NS	NS	NS	NS	X168:X188	0.035	NS	NS
8mso	NS	NS	NS	NS	X196:X188	0.001	NS	NS
I3M	NS	NS	NS	NS	**AOP3:X186**	0.050	X188:X11(ELONG)	0.049
4M-I3M	**AOP3:X11(ELONG**)	0.039	NS	NS	NS	NS	NS	NS
C3	X160:X11(ELONG)	0.009	X120:X11(ELONG)	0.014	**AOP3:X122**	0.001	NS	NS
	X179:X11(ELONG)	0.007	X159:X89	0.023	X122:X89	0.024		
			X159:X11(ELONG)	< 0.001	X122:X112	0.003		
					X89:X11(ELONG)	0.040		
					X89:X171	0.017		
					X89:112	0.014		
					X190:X11(ELONG)	0.006		
					X171:X11(ELONG)	0.031		
					X190:X112	0.011		
					X171:x112	0.015		
C4	**AOP3:X160**	0.027	NS	NS	NS	NS	NS	NS
	X160:X190	0.031						
SC	X179:X11(ELONG)	0.018	X120:X11(ELONG)	0.012	X89:X112	0.028	NS	NS
LC	NS	NS	NS	NS	X196:X188	0.003	NS	NS
Indole	NS	NS	NS	NS	NS	NS	NS	NS

Significant interactions with AOP3 are depicted in bold.

QTL analysis of the Col-0 × Gie-0 UT2 and UT10 populations gave further evidence that in addition to the enzymatic activity, the *AOP3* RNA also influences glucosinolate accumulation. The *AOP3* insertion in the UT2 population was found as a significant QTL for 3msp accumulation indicating that the RNA alone is able to affect the accumulation of this type of glucosinolates (Table 1). We also observed effects on accumulation of other glucosinolates mediated by variation in *AOP3* within the Col-0 × Gie-0 UT10 population. Thus, both UT populations suggested that the *AOP3* RNA affected glucosinolate profiles. QTL mapping in each UT population identified several additional loci involved in controlling glucosinolate levels, among these *GS-ELONG* (Table 2, Figure 6). In the search for loci epistatic with *AOP3* no significant interaction between *AOP3* UT and *GS-ELONG* was found (Table 3). In combination with the epistatic interaction between *AOP3* FL and *GS-ELONG*, this indicates that the regulatory function of the enzymatic activity depends on the state on *GS-ELONG*, but the function of the RNA does not, which is in correspondence with the previous allele-specific interaction for glucosinolate phenotypes (Figure 5).

### Fine-tuning of glucosinolate profiles by the *AOP3* RNA depends on different background loci across populations

To rule out that the effects of the *AOP3* RNA/UT constructs are due to insertion site and assess if any variation in QTL detection amongst the two UT populations are a result of different allele frequencies, we conducted a combined analysis across the two populations. This analysis was based on a model, including the allelic state of *AOP3* UT from the two different positions, significant main effects and interacting loci, as well as a population term. This allows us directly test if a QTL is population dependent or if there were consistent effects of the *AOP3* RNA across both populations and their associated insertion sites.

**Figure 7:**
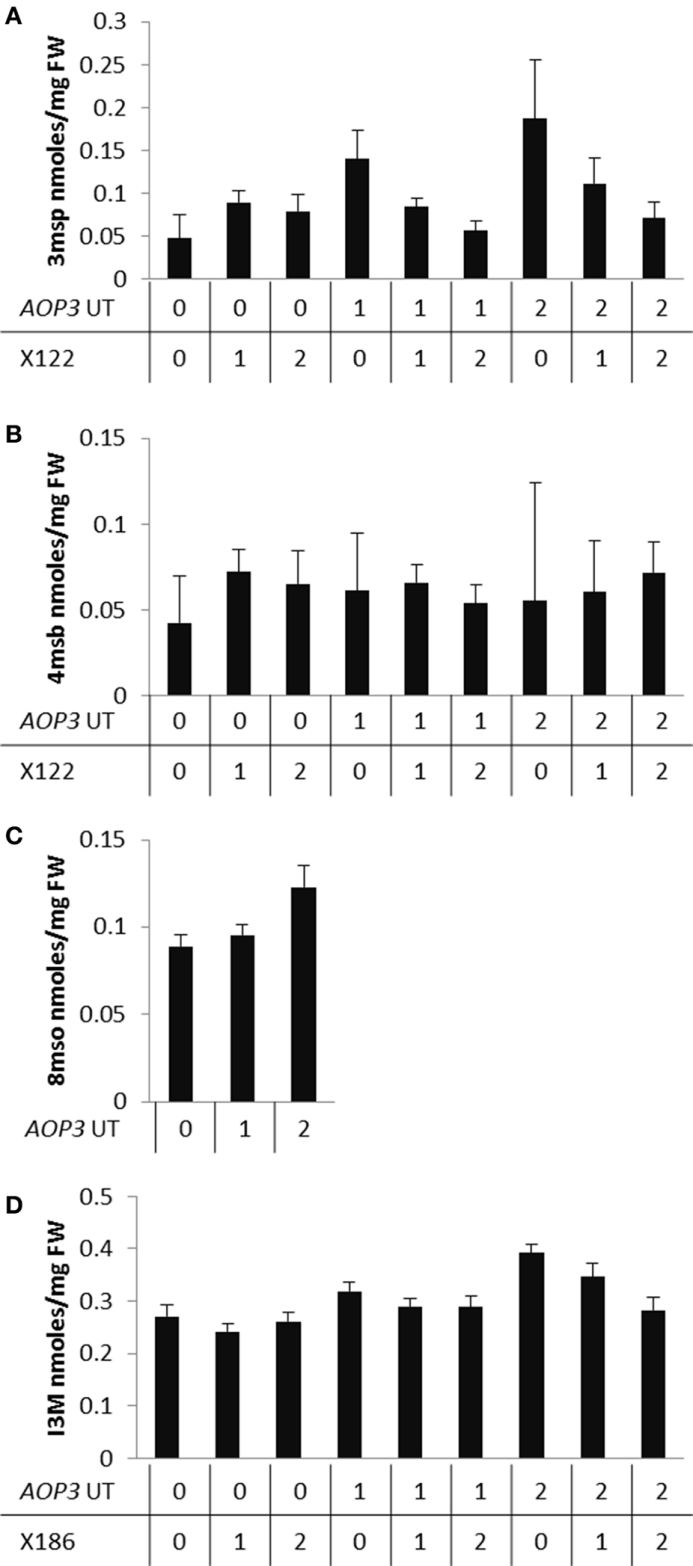
**Allele specific interactions of *AOP3* and QTLs controlling different glucosinolates. (A)** Average leaf levels of 3msp dependent on the genetic state of the interaction of *AOP3* and X122. **(B)** Levels of 4msb dependent on *AOP3* and X122. **(C)** Accumulation of 8mso depends on the state of *AOP3*. **(D)** Levels of I3M controlled by the interaction of AOP3 and X186. “0” indicates homozygous for absence of *AOP3* or the Col-0 allele, “1” heterozygous for *AOP3* or the marker, and “2” homozygous for presence of *AOP3* or the Gie-0 allele.

Based on the results from the mapping in the individual populations, where *AOP3* UT showed epistasis with different QTLs in the UT2 and UT10 populations for different glucosinolates (Table 3), we made combined models only including main effect QTLs and epistatic interactions significant in the pooled UT populations (Table S1). This allowed us to test across different insertion sites and other population effects. The analysis revealed that there is a consistently significant interaction of *AOP3* UT and a locus near marker X122 on chromosome 2 for controlling 3msp accumulation across the populations (Table 4). Allele-specific analysis showed a semi-dominant effect of the presence of *AOP3* and the Col-0 allele for X122 for 3msp (Figure 7). A similar effect is not seen for accumulation of the main C4 glucosinolate, 4-methylsulfinylbutyl glucosinolate (4msb), illustrating the fine-tuning regulatory effect of the *AOP3* RNA. Additionally, the *AOP3* UT construct shows up as significant for controlling the main LC glucosinolate 8-methylsulfinyloctyl glucosinolate (8mso), in the combined populations. However, in this case, no significant interacting loci were found (Table 4). The highest levels of 8mso is found in plants homozygous for *AOP3*, suggesting a dose-dependent effect of the RNA. We also tested for the levels of the indole glucosinolate I3M and found that the *AOP3* RNA also influences the accumulation by interaction with X186 on chromosome 4 (Table 4). The allele-specific interactions showed a semi-dominant pattern of interaction between the *AOP3* RNA and the Col-0 allele of X186.

**Table 4:** **Significance of main effects and interactions with AOP3 RNA in the combined AOP3 UT populations**.

	**AOP3 UT**	
	**Marker**	***P*-value**
3msp	AOP3	NS
	X122	0.015
	X89	NS
	X190	< 0.001
	X171	0.001
	X112	NS
	X120	NS
	X175	NS
	X53	NS
	X16	NS
	X11(ELONG)	< 0.001
	Population	NS
	**AOP3:X122**	**0.008**
	AOP3:population	NS
	X122:population	NS
	**AOP3:X122:population**	**0.027**
4msb	AOP3	NS
	X122	NS
	X89	0.003
	X190	< 0.001
	X171	NS
	X112	< 0.001
	X120	0.021
	X175	NS
	X53	NS
	X16	NS
	X11(ELONG)	0.044
	Population	< 0.001
	AOP3:X122	NS
	X122:population	NS
	AOP3:X122:population	NS
8mso	**AOP3**	**0.024**
	X42	< 0.001
	X13	NS
	X62	0.006
	X190	NS
	X168	NS
	X196	NS
	X16	0.024
	X188	NS
	X11(ELONG)	NS
	Population	< 0.001
	AOP3:population	NS
I3M	**AOP3**	**<0.001**
	X105	0.037
	X188	NS
	X186	0.010
	X120	NS
	X11(ELONG)	< 0.001
	Population	< 0.001
	**AOP3:X186**	**0.008**
	AOP3:population	NS
	X186:population	NS
	AOP3:X186:population	NS

Significant interactions with AOP3 are depicted in bold.

These observations in the combined population of UT2 and UT10 allow us to conclude that the RNA has regulatory functions independent of insertion site, and that it epistatically interacts with different genomic regions than the AOP3 enzyme. In summary different loci including the *AOP3* transgene control the fine-tuning of glucosinolate accumulation, but to fully understand this complex network will require extensive work. We are though able to place both the enzymatic activity and the RNA of *AOP3* as players in the network. The regulatory function of this biosynthetic gene is nevertheless highly dependent on the presence of other regulators and the underlying genomic variation.

## Discussion

We focused on a genetic dissection of the link between the enzyme-encoding gene *AOP3* and glucosinolate accumulation to better understand how natural variation can influence adaptive phenotypes. Using different transgenes and populations, we could explicitly show that *AOP3*, like *AOP2*, has the potential to alter glucosinolate accumulation. We could observe the regulatory function in different F2 populations, where epistasis between *AOP3, GS-ELONG*, and other loci interacted to play a major role for the accumulation of glucosinolates. This was in contrast to our results wherein ectopic expression of *AOP3* in the two accessions varying in *GS-ELONG*, Col-0, and Gie-0, did not change glucosinolate levels. This illustrates the difficulty in understanding the molecular function of a gene on the whole species level. Not only is *AOP3* involved in fine-tuning glucosinolate accumulation and therefore controlling small phenotypic changes, but its effect additionally dependent on the allelic state of the rest of the regulatory network such that the effect is not seen in two chosen accessions, but only in genetic variable F2 populations. This suggests that other regulators might need to be introduced into different networks present in different genotypes to fully understand a gene's function within a species.

The Col-0 × Gie FL6 population showed that the *AOP3* regulatory effect on glucosinolate accumulation is partly explained by the enzymatic activity. The interaction between *AOP3* and *GS-ELONG* showed that this effect was highly dependent on the presence of the 3msp substrate suggesting that this link is largely based on flux. However, it is interesting that in mixed backgrounds from Col-0 and Gie-0, the active AOP3 was able to change the ratio of C3–C4, to an extent not seen among natural accessions (Kliebenstein et al., 2001b; Kroymann et al., 2003). Based on the QTL mapping, we suggest that this may depend on the loci showing significant interactions with *AOP3*, and these loci vary from those in Ler-0. In Col-0 × Gie-0 population, the observed ratio suggests that AOP3 is able to compete with MAM1 and pull glucosinolates out of the elongation cycle at the C3 step, which is surprising as AOP3 is several enzymatic steps downstream of the MAM-catalyzed C3–C4 step. Further, AOP3 is thought to localize to the cytosol while MAM1/2 localize in the chloroplast (Sønderby et al., 2010) ruling out any possible physical interactions to mediate this flux diversion. It is therefore interesting to conduct follow-up studies to assess if this is truly a pull and depletion of substrate and products through the pathway or if other regulatory mechanisms are mediating this distinct change in the glucosinolate profile.

In addition to the functional AOP3 influencing glucosinolate accumulation, we obtained evidence that the *AOP3* RNA in the form of the *AOP3* UT construct can separately contribute to the regulation of glucosinolate production. QTL mapping in the Col-0 × Gie-0 UT populations indicated that the RNA-expressing UT construct had an effect on the glucosinolate accumulation. Interestingly, the RNA-expressing UT and the enzyme-expressing FL constructs affected different aliphatic glucosinolates and epistatically interacted with different background loci. This may suggest that competing regulatory roles of the RNA and enzyme and that the RNA-associated effect is separable from the enzyme-mediated effects. Thus, multiple molecular components of the naturally variable *AOP3* gene influence glucosinolate accumulation. The RNA might be processed into a small RNA and mediate a regulatory function at for instance the DNA level or interact with proteins in the network to influence glucosinolate biosynthesis, and thereby change the output of the glucosinolate regulatory network. This adds to the discussion of whether many of the characterized naturally variable genes, have functions that still remain unknown.

This study documents that a regulatory network cannot be fully described only by identifying the genes involved. A regulatory network consists of many different kinds of molecules; metabolites, RNAs, proteins, and enzymes that all interact which each other to fine-tune the metabolism and the phenotype of a plant. The consequence of *AOP3* expression varies to a large extent dependent on the polymorphic genetic background. The identified epistatic loci for the enzymatically active AOP3 include the *GS-ELONG* locus decisive for the substrate availability. For the *AOP3* RNA, the QTLs may encode a physical interaction partner, a direct target, or a more distantly connected component of glucosinolate regulatory networks. Our findings allow us to expand the knowledge on glucosinolate regulation showing that *AOP3* affects glucosinolate levels under certain conditions and establish *AOP3* as a multifunctional gene both encoding an enzyme changing the flux through the pathway, but also an RNA involved in fine-tuning of glucosinolate profiles.

### Conflict of interest statement

The authors declare that the research was conducted in the absence of any commercial or financial relationships that could be construed as a potential conflict of interest.
