# Supplementary material for: Investigation of the multifunctional gene AOP3 expands the regulatory network fine-tuning glucosinolate production in Arabidopsis
Source: Front Plant Sci. 2015 Sep 23;6:762. doi: 10.3389/fpls.2015.00762 (PMC4585220; doi:10.3389/fpls.2015.00762)
Supplement: Supplementary file 2 [file DataSheet2.DOCX]

**File S1 Models for QTLs controlling different glucosinolate levels**

Models were generated based on WinQTLCartographer Version 2.5_011 results and used for all types for SC glucosinolates, LC glucosinolate or indolic glucosinolates in the different populations using R. For the combined UT population models were generated for specific glucosinolates and the effect of the population was included. Interactions are depicted with “:”.

**Models for FL6 population**

SC ~ XAOP3 + X160 + X190 + X179 +X11 + XAOP3:X160 + XAOP3:X190 + XAOP3:X179 + XAOP3:X11 + X160:X190 + X160:X179 +X160:X11 +X190:X179 +X190:X11 + X179:X11

LC ~ XAOP3 + X196 + X66 + X11 + XAOP3:X196 + XAOP3:X66 + XAOP3:X11 + X196:X66 + X196:X11 +X66:X11

INDOL ~ XAOP3 + X164 + X11 +XAOP3:X164 + XAOP3:X11 + X164:X11

**Models for FL9 population**

SC ~ X29 + X120 + X159 + X170 +X172 + X89 + X11+ X29:X120 + X29:X159 + X29:X170 +X29:X172 + X29:X89 + X29:X11 +X120:X159 + X120:X170 +X120:X172 + X120:X89 + X120:X11 +X159:X170 +X159:X172 + X159:X89 + X159:X11 +X170:X172 + X170:X89 + X170:X11 +X172:X89 + X172:X11 + X89:X11

LC ~ X29 + X120 + X159 + X175 +X47 + X11 + X29:X120 + X29:X159 + X29:X175 + X29:X47 + X29:X11 + X120:X159 + X120:X175 + X120:X47 +X120:X11 +X159:X175 +X159:X47 +X159:X11 +X175:X47 +X175:X11 +X47:X11

INDOL ~ X29 + X49 + X147 + X42+ X89+ X11 + X29:X49 + X29:X147 + X29:X42 + X29:X89 + X29:X11 + X49:X147 + X49:X42 +X49:X89 +X49:X11 +X147:X42 +X147:X89 +X147:X11 +X42:X89 +X42:X11 +X89:X11

**Models for UT2 population**

SC ~ X61 + X122 + X89 + X11 + X190 + X171 + X112 + X61:X122 + X61:X89 + X61:X11 + X61:X190 + X61:X171 + X61:X112 + X122:X89 + X122:X11 + X122:X190 + X122:X171 + X122:X112 +X89:X11 + X89:X190 + X89:X171 + X89:X112 + X11:X190 + X11:X171 + X11:X112 +X190:X171 + X190:X112 + X171:X112

LC ~X61 +X168 +X196 +X16 +X188 +X11 +X61:X168 +X61:X196 +X61:X16 +X61:X188 +X61:X11 +X168:X196 +X168:X16 +X168:X188 +X168:X11 +X196:X16 +X196:X188 +X196:X11 + X16:X188 +X16:X11 +X188:X11

INDOL ~ X61 +X120 +X186 +X188 +X11 +X61:X120 +X61:X186 +X61:X188 +X61:X11 +X120:X186 +X120:X188 +X120:X11 +X186:X188 +X186:X11 +X188:X11

**Models for UT10 population**

SC ~ X160 + X120 + X175 + X53+ X16 + X11 +X160:X120 + X160:X175 + X160:X53+ X160:X16 + X160:X11 +X120:X175 + X120:X53+ X120:X16 + X120:X11 +X175:X53 + X175:X16 + X175:X11 +X53:X16 + X53:X11 + X16:X11

LC ~ X160 +X42 +X13 +X62 +X190 +X160:X42 +X160:X13 +X160:X62 +X160:X190 +X42:X13 +X42:X62 +X42:X190 +X13:X62 +X13:X190 +X62:X190

INDOL ~ X160 +X105 +X188 +X11 +X160:X105 +X160:X188 +X160:X11 +X105:X188 +X105:X11 +X188:X11

**Models for combined UT populations**

3msp/4msb ~ X61.X160 +X122+X89+X190+X171+X112 + X120 + X175 + X53+ X16 + X11 + Population +X61.X160:Population +X61.X160:X122 + X122:Population +X61.X160:X122:Population

8mso ~ X61.X160+X42+X13+X62 +X190+X168+X196+X16+X188+X11+Population+X61.X160:Population

I3M ~ X61.X160 +X105 +X188 +X11 +X120 +X186 +Population +X61.X160:Population +X186:Population + X186:X61.X160 + Population:X186:X61.X160
