# Supplementary material for: Investigation of the multifunctional gene AOP3 expands the regulatory network fine-tuning glucosinolate production in Arabidopsis
Source: Front Plant Sci. 2015 Sep 23;6:762. doi: 10.3389/fpls.2015.00762 (PMC4585220; doi:10.3389/fpls.2015.00762)
Supplement: Supplementary file 3 [file Image1.PDF]

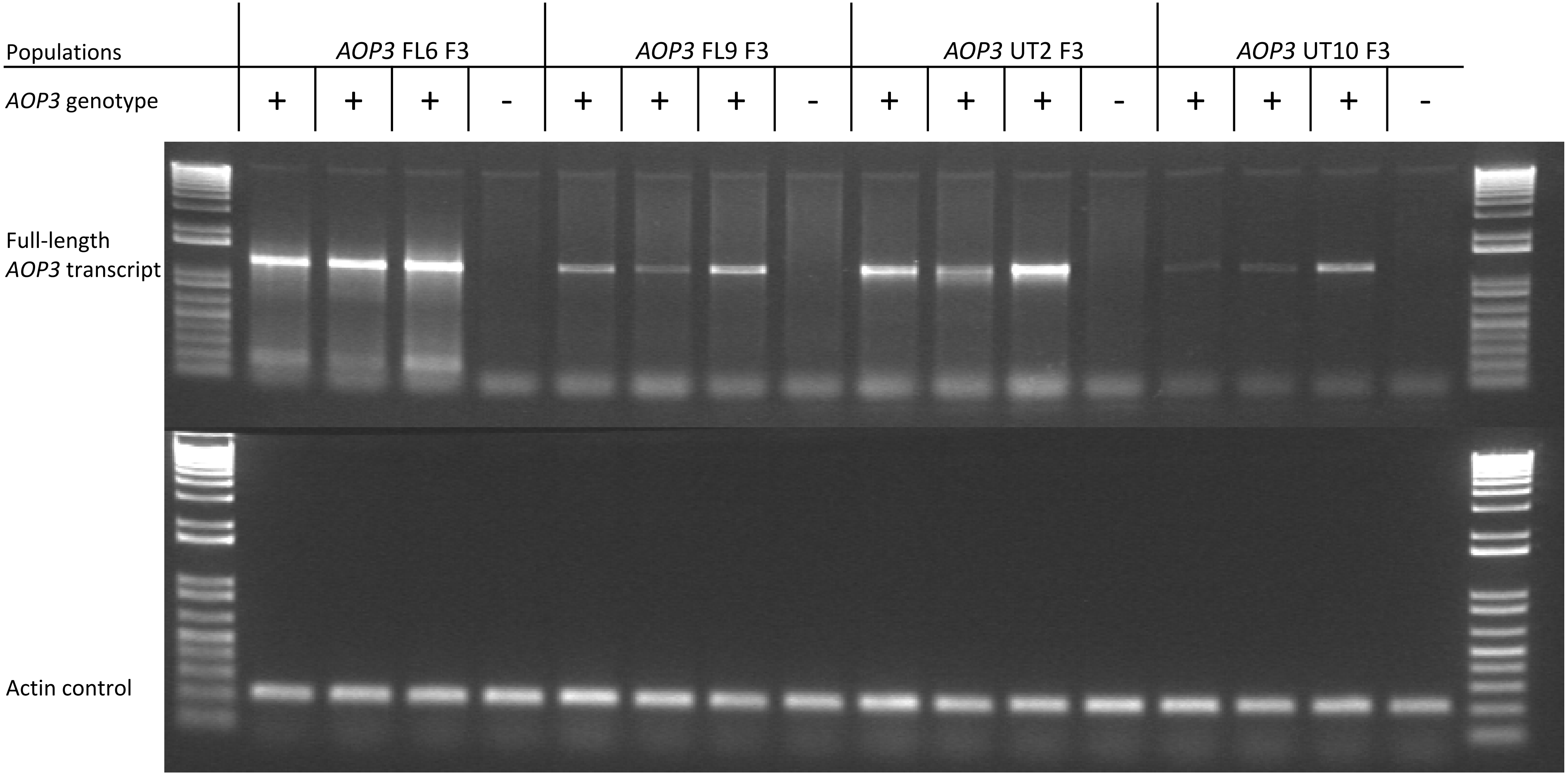

Figure S1 Expression of *AOP3* transcripts in F3 generation
From the four different mapping populations F2 plants were genotyped for presence of absence of the inserted *AOP3*. Four F2 plants per population were picked for expression analysis in the subsequent generation; three positive for *AOP3* and one negative for *AOP3* insert. RT-PCR on pools of the F3 seedlings show expression of full-lenght *AOP3* (~1950 bp) from F3 plants carrying the transcript, whereas no expression was detected in the plants negative for insert. Furthermore, actin transcript (~230bp) was detected in all samples.
